# An Overview on Primary Sclerosing Cholangitis

**DOI:** 10.3390/jcm9030754

**Published:** 2020-03-11

**Authors:** Cătălina Vlăduţ, Mihai Ciocîrlan, Dana Bilous, Vasile Șandru, Mădălina Stan-Ilie, Nikola Panic, Gabriel Becheanu, Mariana Jinga, Raluca S. Costache, Daniel O. Costache, Mircea Diculescu

**Affiliations:** 1 Department of Gastroenterology, Prof Dr Agrippa Ionescu Clinical Emergency Hospital, 7000 Bucharest, Romania; drcatalinavladut@gmail.com; 2 Carol Davila University of Medicine and Pharmacy, 7000 Bucharest, Romania; d_bilous@yahoo.com (D.B.); m_ilie2001@yahoo.com (M.S.-I.); mariana_jinga@yahoo.com (M.J.); mmdiculescu@yahoo.com (M.D.); 3 Department of Gastroenterology, Clinical Emergency Hospital Bucharest, 7000 Bucharest, Romania; sandru_vasile@yahoo.com; 4 Dr. Dragisa Misovic-Dedinje University Clinic, 11000 Belgrade, Serbia; nikola.panicmail@gmail.com; 5 Department of Anatomopathology, Fundeni Clinical Institute, 7000 Bucharest, Romania; gbecheanu@yahoo.com; 6 Department of Gastroenterology, Carol Davila University Central Emergency Military Central Hospital, 7000 Bucharest, Romania; 7 Department of Dermatology, Carol Davila University Central Emergency Military Central Hospital, 7000 Bucharest, Romania; daniel_costache@yahoo.com; 8 Department of Gastroenterology, Fundeni Clinical Institute, 7000 Bucharest, Romania

**Keywords:** primary sclerosing cholangitis, cholangiography, choleangiectasias, liver transplant

## Abstract

Primary sclerosing cholangitis is a progressive liver disease characterized by chronic inflammation leading to liver fibrosis and cirrhosis. Even though the exact pathogenesis is still unclear, a combination of autoimmune, environmental, and ischemic factors could explain certain aspects of the disease. The most important diagnostic step is cholangiography, which can be obtained either by endoscopic retrograde cholangiopancreatography (ERCP), magnetic resonance cholangiography (MRCP as the gold standard), or percutaneous transhepatic cholangiography. It shows multifocal short biliary duct strictures leading to the “beaded” aspect. Cholangiocarcinoma and colorectal adenocarcinoma are the most feared complications in patients with Primary sclerosing cholangitis (PSC). Continuous screening consists of annual clinical, biochemical, and ultrasound assessments in asymptomatic patients and annual colonoscopy in patients with PSC and inflammatory bowel disease. In newly diagnosed patients with PSC, colonoscopy is mandatory and, if negative, then, a repeat colonoscopy should be performed in 3–5 years. The lack of efficient curative medical treatment makes invasive treatments such as liver transplant and endoscopy the mainstream for managing PSC and its complications. Until now, even though only ursodeoxycholic acid has shown a moderate clinical, biochemical, and even histological improvement, it has no significant influence on the risk of cholangiocarcinoma, liver transplant need, or death risk and it is no longer recommended in treating early PSC. Further studies are in progress to establish the effect of molecular-targeted therapies in PSC.

## 1. Introduction

Primary sclerosing cholangitis (PSC) is a chronic liver disease with progressive inflammation, liver fibrosis, and multifocal biliary duct stenosis. The first description of the disease was made in 1924 by Delbet, and after the discovery of endoscopic retrograde cholangiopancreatography (ERCP), more concise information helped us to understand the disease [1]. The pathogenesis remains elusive though different theories have been proposed; the key player remains the lack of protection against the bile acids toxicity and autoimmunity [2]. Small duct PSC is a variant of the disease with typical cholestatic and liver biopsy suggestive of PSC but normal cholangiography. Association with other autoimmune diseases such as autoimmune hepatitis or pancreatitis, diabetes mellitus type 1, Graves disease, or multiple sclerosis can occur. Association with inflammatory bowel disease is frequent. Distinction from choledocholithiasis, biliary surgery or trauma, bacterial cholangitis, acquired immunodeficiency syndrome, infiltrative liver diseases, and pancreatic or biliary cancer is made through the term “primary” [3]. In most of the cases, the diagnosis is now established through magnetic resonance cholangiography (MRCP) showing multifocal strictures and saccular dilatations of the bile ducts (“beaded” appearance) [4]. Recently, diagnosis of PSC is made by cholestatic liver biochemistry with typical cholangiographic aspect in the absence of other causes of secondary sclerosing cholangitis [5].

## 2. Epidemiology

Recent studies show that PSC has an overall incidence rate of 0.77 per 100,000 person-years. Most patients with PSC range between 25 and 45 years old, with a median age at diagnosis of 41 years [1]. Even though 90% of patients with PSC have inflammatory bowel disease (IBD), only 2.4–4% of patients with ulcerative colitis and 1.4% of patients with Crohn’s disease have PSC [1]. Approximately 60–70% of patients with PSC and ulcerative colitis (UC are men, though in patients without UC, there is a slight female predominance (female:male ratio = 1:0.8). The association with hepatobiliary malignancies and colorectal cancer is firmly established: 10.9% of patients with PSC have biliary cancer. Colorectal adenocarcinoma risk is fivefold higher in patients that have UC and PSC [4]. The prevalence of PSC is increased by genetic factors (11-fold risk for a sibling with first-degree relatives with PSC), nonsmoking (probably associated with ulcerative colitis), and other autoimmune diseases (25% of patients with PSC) [3,6]. Twenty-five percent of patients with PSC present gallstones as part of the disease, not as exclusion criteria. Gallbladder masses or polyps are encountered in 4% to 6.5% of patients with PSC, more than half being malignant [4]. Gallbladder polyps over 8 mm are an indication for cholecystectomy in patients with PSC [7].

## 3. Pathogenesis

**Figure 1:**
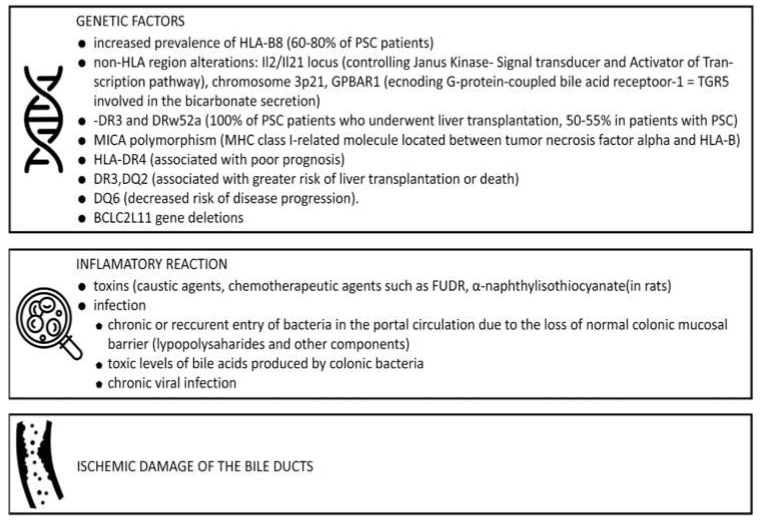
Factors involved in etiopathogenesis of Primary sclerosing cholangitis (PSC) [1,6].

To date, the etiopathogenesis of PSC is still unknown, even though predisposing genetics, environmental factors, and circulatory dysfunction play key roles (Figure 1) [4].

Protection against the bile acids is usually ensured by the bicarbonate “umbrella” generated by the Na^+^-independent Cl/HCO_3_ anion exchanger (AE2) and active Cl-transporters, most notably the ATP-driven cystic fibrosis transmembrane conductance regulator (CFTR) and the Ca^++^-driven anoctamin 1 channel. In PSC, multiple disturbances in the bile homeostasis appear, such as mutations in CFTR. Through this mechanism, the potential benefit of therapies with ursodeoxycholic and non-ursodeoxycholic acid is explained. Research on other mechanisms involved in bile homeostasis (farnesoid X receptor, retinoid X receptor, peroxisome proliferator-activated receptor-alpha, and pregnane X receptor) is a hot topic and requires further research [1]. The gut–liver axis component might be essential in the pathology of PSC since early studies show that gut leakage leads lipopolysaccharides (LPS) to cross the colonic membrane, to reach the liver, and to interfere with the immune system through toll-like receptor signaling. Gut-derived antigens can be solely responsible for the immune response and activation of T cells and then B cells. This leads to the secretion of antibodies such as antinuclear antibodies (ANA), smooth muscle antibodies (SMA) (13–20%), and atypical perinuclear antineutrophil cytoplasmatic antibodies (ANCA) (65–88%) [1,8]. Recent studies have identified novel target molecules involved in the pathogenesis of PSC—vascular adhesion protein 1 (VAP-1) and lysyl oxidase like-2 (LOXL2)—and are important in dysbiosis in gut microbiota [9].

Typically, in PSC, we encounter T-cell immune-mediated attacks towards bile duct epithelial cells. The predominant cell present in the inflammatory infiltrate is the T cell (increased CD4+ in the liver and decreased circulating CD8+ cytotoxic cells), followed by macrophages and neutrophils (both found in the portal areas). Typically, we encounter an increased number of γδ T cells, both in portal liver areas and in peripheral blood with the overrepresentation of Vβ3 T-cell receptor gene segments in hepatic T-cell populations, though not in peripheral blood. Persistent stimulation of the inflammatory response leads to multiple interactions with hepatic stellate cells (leading to fibrosis and cirrhosis) and portal myofibroblasts (leading to multiple stenoses of the bile ducts) [10]. However, there is an increase in the level of circulating immune complexes and deficient clearance of immune complexes. The risk of malignancy can be explained by the constant exposure to bile acids during chronic cholestasis, along with the effect of various molecules of chronic inflammation and regeneration such as interleukin 6 and WNT signaling. Other immunological alterations are serum elevation of IL-8 and IL-10 (exaggerated humoral immunity), abnormal antigen expression of bile duct epithelial cells, cross-reacting with colonic epithelial cells, aberrant expression of human leukocyte antigen (HLA) class II antigens and intercellular adhesion molecule (ICAM)-1 on ductal epithelial cells, and mutation in the gene encoding CTFR [1,3].

Even though the molecular mechanism for the development of cholangiocarcinoma in patients with PSC is not fully understood, pro-oncogenic processes due to chronic biliary inflammation and bile stagnation alongside cocarcinogenic stimuli due to environmental and genetic factors are to blame. The sequence of carcinogenesis follows certain steps: inflammation, biliary metaplasia, low-grade dysplasia, high-grade dysplasia, and cholangiocarcinoma. Carcinogenesis is represented by the activation of stem cell niche within the peribiliary glands and expansion of the peribiliary vascular plexus. Cellular aspects show a transition from epithelial to mesenchymal features, absence of primary cilia, and increase in autophagy and senescence. Moreover, secretion of tumor necrosing factor (TNF-α) and nitric oxide might facilitate oncogenesis due to DNA damage, cholangiocyte proliferation, and inhibition of apoptosis. Recent studies show that NKG2D polymorphism is a risk factor for oncogenesis [11].

## 4. Natural History and Prognostic Models

Most of the studies from the literature show that PSC is a progressive disease leading to liver failure and death, even though each patient has its disease course (Table 1). The median time to progress from diagnosis to death or liver transplantation is estimated at 9 to 18 years [7].

To simplify these variations, different prognostic models have been developed in time, the revised Mayo Clinic Risk Score being the most frequently used in medical practice. It is a noninvasive score and is dependent on age, bilirubin, aspartate aminotransferase and albumin levels, and the presence of variceal bleeding. The Model for End-Stage Liver Disease (MELD) score is used in transplant settings, predicting 3-month mortality in end-stage liver disease. Child–Pugh–Turcotte and King’s College scores are of small use in PSC. Other invasive scores that are useful in selected cases are APRI (aspartate aminotransferase to platelet ratio index), Fib, Enhanced Liver Fibrosis ELF score, liver stiffness (noninvasive tests), Amsterdam score (based on intrahepatic and extrahepatic changes on cholangiography), Ludwigand Batt’s, Nakanuma, and Ishak scores (histology scores) [2]. Most deaths are attributable to cholangiocarcinoma (58%), liver failure (30%), and variceal bleeding (9%) [5].

## 5. Diagnosis

### 5.1. Signs and Symptoms

Even though patients with PSC are generally asymptomatic (15–44% of patients at the time of diagnosis), signs and symptoms in PSC range from fatigue to extreme pruritus (Table 1) [1,4]. The previous history of IBD and hepatocytolysis should be taken into consideration, and prompt evaluation to exclude PSC should be the next step [12]. Hepatobiliary or colonic malignancy might complicate the disease course [2].

### 5.2. Laboratory Tests

Laboratory tests hallmark in PSC represents a cholestatic profile: increase of alkaline phosphatase (ALP) 3 to 5 times normal, even though up to 6% of patients can present normal ALP levels. Seric transaminases can be increased, though under 4–5 times normal (except in the pediatric population and overlap syndrome). Bilirubin can be normal or can fluctuate in time, and higher values can be a marker of poor prognosis. Low albumin can show decompensated disease with hepatic synthetic dysfunction, malnutrition, or active IBD [2,5].

### 5.3. Immunological and Serological Markers

Immunological and serological markers can be present, such as hypergammaglobulinemia (encountered in almost 30% of patients), increased serum immunoglobulin M (40–50% of patients with PSC), HLA DRx52a, and positive perinuclear anti-neutrophil cytoplasmic antibodies (pANCA). The last is referred to as ANNA (antineutrophil nuclear antibodies) and represents the most commonly found serological marker in 30% to 80% of cases. Unlike other autoimmune diseases, the level of pANCA is not a marker of severity or prognosis for PSC. Positive ANA (in 24–53% of patients with PSC), SMA (13–20% of patients with PSC), anticardiolipin antibodies (up to 66% of cases), and rheumatoid factor; increased hepatic and urinary copper; and decreased serum ceruloplasmin may be encountered in PSC, though are not specific [1]. Antimitochondrial antibodies are usually absent in PSC and may guide the diagnosis toward primary biliary cholangitis. High IgG4 levels might be a sign of rapidly progressive disease in non-treated patients, positive in up to 10% of cases or IgG4-associated disease (using the HISORt criteria – Histology, Imaging and Serology, Response to Steroids); therefore, all patients should be tested for IgG4 at least once [3]. The cutoff value of carbohydrate antigen (CA) 19–9 is considered 129 U/mL. However, this is dependent on the expression of Lewis antigen (10% of the population lacks Lewis antigen) and on the genotypic variants of fucosyltransferase 2 and 3 (improves tumor marker sensitivity) [7].

### 5.4. Imaging

**Figure 2:**
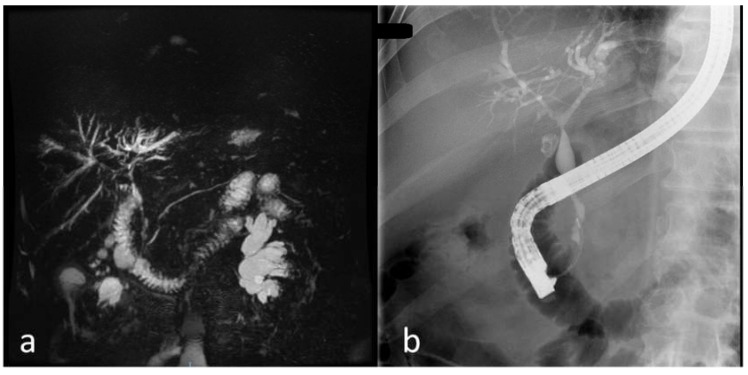
Cholangiography of a patient with PSC from our clinical practice: (**a**) magnetic resonance cholangiography (MRCP) images; (**b**) endoscopic retrograde cholangiopancreatography (ERCP) images.

Imaging of the biliary tract is the most important step in the diagnosis, with PSC involving the entire biliary tree heterogeneously. If the diagnosis is clear after the imagistic aspect, there is no need for a liver biopsy. Ultrasound is not very accurate, though it can show thickening of the common bile duct (CBD) wall or focal bile duct dilatations. Complications of PSC such as gallstones (present in 25% of cases), cholecystitis, mass lesions in the gallbladder or CBD, cirrhosis (marked hypertrophy of the right and caudate lobes with atrophy of the rest of the liver) can be diagnosed with ultrasound; therefore, since it is more accessible and cheaper than MRCP, it should be taken into consideration beforehand. CT might show thickened walls of the bile ducts, saccular dilatations of the CBD, mass lesions, or evidence of portal hypertension. If obtained by MRCP, PTC, or ERCP, cholangiography remains the most specific examination in PSC. MRCP is the test of choice since it is noninvasive and it shows the typical “beaded” aspect of CBD due to multifocal, short, circumferential strictures alternating with normal aspect CBD or mildly dilated segments (Figure 2) [2,5].

Long strictures raise the suspicion of cholangiocarcinoma and should further be investigated by ERCP and ductal sampling. It is essential to exclude secondary causes of sclerosing cholangitis. In early PSC, only ulcerations can be present; therefore, ERCP is essential in the diagnosis of the disease in the early state. Most frequently, both intra- and extrahepatic bile ducts present specific changes for PSC. However, in 11% of cases, strictures are present only in the intrahepatic ducts and, in 2% of cases, strictures appear only in the extrahepatic ducts. Moreover, the gallbladder and cystic duct might present changes (masses in the gallbladder) [3]. ERCP and concomitant ductal sampling by brush cytology or endobiliary biopsies are essential in dominant strictures or suspected cholangiocarcinoma. Amsterdam classification in PSC is used to classify intrahepatic (stages 0 to III) and extrahepatic (stages 0 to IV) cholangiographic changes [13]. Dynamic three-dimensional biliary scintigraphy (99mTc-HIDA SPECT) is used to differentiate parenchymal and ductal function in the abnormal liver function [6]. Endoscopic ultrasound with biopsies is essential and more accurate than MRIs or CTs in diagnosing hilar and distal cholangiocarcinoma or regional lymph nodes for staging cholangiocarcinoma [14]. A recent development in endoscopy units offered clearer and more accurate views with peroral cholangioscopy (single operator cholangioscopy—SpyGlass), allowing direct visualization of the extrahepatic bile duct strictures and the possibility to do biopsies [13]. Ultrasound endoscopy can be used in the differential diagnosis of distal dominant strictures. However fine-needle aspiration of perihilar biliary strictures in liver transplant candidates is not recommended due to the tumor seeding risk [7].

### 5.5. Histology

**Figure 3:**
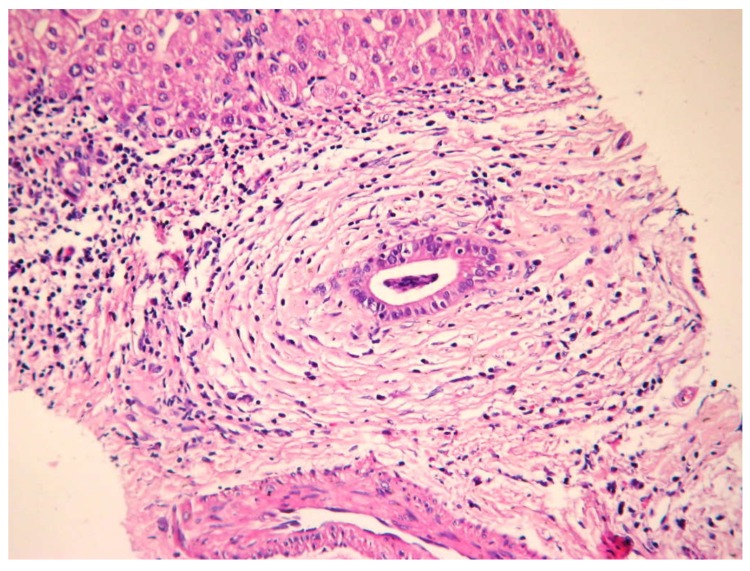
Portal space with periductal fibrosis and moderate inflammation hematoxylin eosin (HE) 200×.

**Figure 4:**
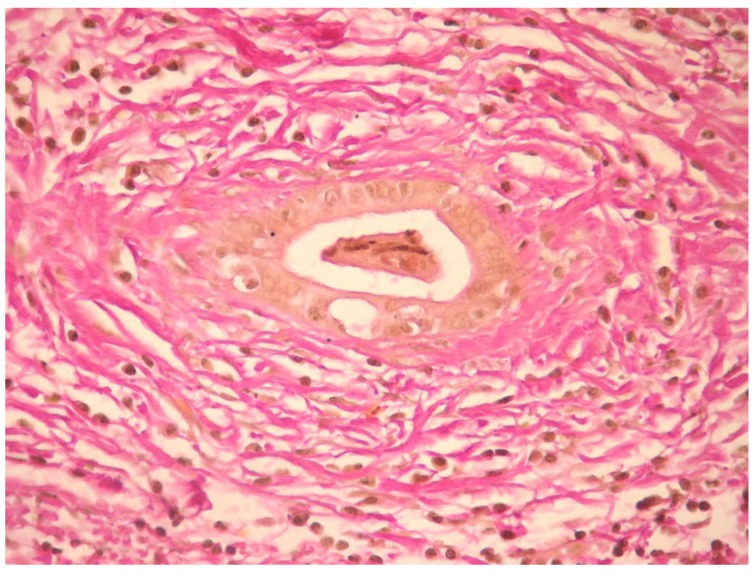
Periductal fibrosis—Van Gieson coloration 400×.

Histology specimens were diffusely thickened, with the fibrotic wall having a mixed inflammatory infiltrate (lymphocytes, plasma cells, and neutrophils) that affects both the epithelium and biliary glands. Studies showed that florid hyperplasia of the biliary glands, neural and bile duct proliferation, periductal inflammation, and ductopenia can also be seen on biopsy specimens. The beaded aspect as seen on MRCP is explained histologically by choleangiectasias [1]. The pathognomonic lesion in PSC is a fibro-obliterative process leading to “onion skin” scar due to the concentric layers of fibrosis that form around the bile duct, though it is present only in 25% of cases (Figure 3 and Figure 4). Complete obliteration of the small interlobular and septal bile duct branches results in fibro-obliterative cholangitis and can be present in 5–10% of biopsy specimens [3]. 

Histologic examination allows us to use a staging system as follows: stage I—portal stage: inflammation confined to the portal tracts, with connective tissue expansion, cholangitis but no fibrosis; stage II—periportal stage: inflammation that extends beyond the limiting plate leading to interface hepatitis (“piecemeal necrosis”) and fibrosis in the portal and periportal areas; stage III—septal stage: septal or bridging fibrosis sometimes associated with bridging necrosis (though uncommon); and stage IV—cirrhosis: complete septal fibrosis, sometimes associated with nodular regeneration [2,12].

### 5.6. Noninvasive Tests for Assessing Liver Fibrosis

Transient elastography and serological tests for assessment of liver fibrosis are more commonly used in staging liver fibrosis and predict prognosis in patients with PSC. Magnetic elastography is gaining more and more importance as a noninvasive test for liver fibrosis [5].

## 6. Differential Diagnosis

Differential diagnosis includes other causes of cholestatic syndrome such as structural causes, mostly excluded by ultrasound endoscopy or cross-sectional images: choledocholithiasis, pyogenic hepatic abscesses involving the bile ducts, systemic fungal infections, acquired immunodeficiency syndrome cholangiopathy, choledochal cyst, cystic fibrosis, intrahepatic cholangiocarcinoma (external compression on CBD), cholangitis glandularis proliferans or eosinophilic cholangitis, papillary tumors, histiocytosis X, Caroli’s disease, congenital hepatic fibrosis (traction effect on bile ducts), and autosomal recessive polycystic kidney disease (mass effect on bile ducts). Liver biopsy is not mandatory but is useful in staging and excluding other causes of chronic cholestasis or autoimmune hepatitis, primary biliary cirrhosis (PBC). The last two resemble PSC at diagnosis but PBC affects women (9 times more frequent in women) and is rarely associated with IBD, with elevated AMA and “florid duct lesions” in the histopathological view. Autoimmune hepatitits (AIH) presents elevated serum aminotransferases compared to bilirubin and phosphatase, ANA- or ASMA-positive panels, biopsies with extensive lymphoplasmacytic inflammation, and normal cholangiography [2,12]. ABCB4/MDR3 deficiency has a unique cholangiography with large unifocal or multifocal spindle-shaped intrahepatic bile duct dilatations but no bile duct stenosis. The diagnosis is suspected in cholecystectomies patients around 40 years old with associated cholestasis and is confirmed by ABCB4 genotyping [13].

## 7. Disease-Related Complications

Disease-related complications include fatigue (60–70% of patients) correlated with the disease severity and pruritus (40–60% of cases), which is independent of histological stage even though extrahepatic stenosis can influence symptom intensity (leading to sleep disturbances, emotional/psychological distress, impaired quality of life, and even suicidal ideation). Pruritus is usually aggravated at night and can be exacerbated by heat and pregnancy. Medical treatment of pruritus is important in improving patient’s quality of life (QoL), and treatment strategies start with emollients and antihistamines, cholestyramine (first-line treatment of pruritus), colestipol hydrochloride, or colesevelam. If there is no improvement under this treatment, rifampin or opiate agents such as naloxone or naltrexone should be taken into consideration as second-line treatment. The third line of treatment after endoscopic therapy of the dominant stricture is plasmapheresis or even liver transplant. Metabolic bone disease is a possible complication (also known as hepatic osteodystrophy) present as osteopenia (~50% of patients) rather than osteomalacia, osteopenia due to the altered calcium and vitamin D metabolism (deficient), and steatorrhea (due to the altered gut–liver axis or associated celiac disease or exocrine pancreatic insufficiency). Bone mineral density screening with dual X-ray absorption at diagnosis and every 2–4 years is mandatory. Micronutrient deficiency could be encountered (fat-soluble vitamins malabsorption due to the impaired bile acid delivery system): vitamin A deficiency leading to impaired night vision (20–50% of cases), vitamin E deficiency leading to ataxia, and vitamin K deficiency leading to coagulopathy (treatment of 5 mg/day daily with repeated prothrombin time) [5,14]. All malabsorption cases should be treated with oral supplementation or even parenteral route in severe intestinal fat malabsorption. In vitamin A deficiency with night blindness treatment with 25,000–50,000 IU two-three times a week is essential. Oral replacement in vitamin E deficiency is important even in asymptomatic patients [2,14]. In patients with PSC in need of corticotherapy for associated IBD or liver transplantation and with a risk of bone disease, daily vitamin D 400 IU (10 µg) and calcium supplements are mandatory [5]. 

Biliary calculi are most frequently pigmented and can be present in both intrahepatic and extrahepatic bile ducts and gallbladder, leading to pain and recurrent bacterial cholangitis. These require immediate treatment: ERCP in most cases and surgery in complex cases. Dominant strictures defined as stenosis with a diameter of less or equal than 1.5 mm in CBP or 1 mm in the intrahepatic duct within 2 cm from the hepatic confluence can appear in 15–20% of patients, clinically manifested with sudden jaundice or symptoms of bacterial cholangitis (the most frequent complication of PSC) and diagnosed with cholangiography (preferably ERC due to potential therapeutic procedures—discussed in the treatment section). The previous ERCP is a potential risk factor for cholangitis, especially if stents are being placed. Most commonly described bacteria in cholangitis are Escherichia coli, Klebsiella, Enterococcus, Clostridium, Streptococcus, Pseudomonas, and Bacteroides species. Only 12% of cases presented Candida species in bile cultures, though the clinical relevance is still unknown, and antifungal treatment should be taken into consideration only if other antibiotics fail [5]. Antibiotherapy in bacterial cholangitis is essential, consisting in a 5–7 days course of treatment with broad-spectrum antibiotic (Fluoroquinolone (usually used as first-line), cephalosporin (usually used in more severe cases), and beta-lactamase inhibitor), sometimes adding antibiotics that cover anaerobes. In recurrent cholangitis, rotational antibiotics, every 3–4 weeks, is mandatory o reduce the risk of resistance [5,13]. 

Cholangiocarcinoma can occur in 7–13% of patients with PSC and involves the hilum (75% of cases), intrahepatic ducts (16% of cases), and gallbladder (8% of cases). Moreover, 30% of patients with PSC at the time of liver transplant are diagnosed with cholangiocarcinoma. Even though the diagnosis is challenging due to the similarities with typical stenosis encountered in PSC, cholangiography, elevated CA19-9 (over 100 U), endoscopic biopsy, and cytological brushing are important. Positron emission tomography (PET-CT) and fluorescence in situ hybridization (FISH) are showing great promise in the diagnosis of cholangiocarcinoma in PSC patients, though not for screening. FISH uses labelled DNA probes to detect abnormal loss or gain of selected chromosomes or chromosome foci in individual cells, and the presence of serial or multifocal polysomy represent increased risk of cholangiocarcinoma [3,11]. Serologic and biliary biomarkers such as proteomic profiling and microRNAs and advanced endoscopic imaging techniques (intraductal probes for endoscopic ultrasonography (EUS), confocal laser microscopy, and narrow-band imaging cholangioscopy with site-directed biopsies) are still under investigation but can lead to a more accurate and easy diagnosis in the future. Testing for specific methylation patterns (CDO2, CNRIP1, SEPT9, and VIM) using DNA extracted from biliary is a potential marker for malignancy and can be used in association with FISH in diagnosing cholangiocarcinoma [11]. Treatment options for cholangiocarcinoma in PSC are rather limited: liver transplant and resection being indicated in perihilar masses under 3 cm with prior neoadjuvant external beam radiation and radiosensitizing chemotherapy, endoscopic brachytherapy, and oral capecitabine before exploratory laparoscopy to ensure the curative potential of the surgery. In patients with unresectable, perihilar, early-stage I–II cholangiocarcinoma, palliative treatment should be considered: chemotherapy using gemcitabine combined with cisplatin, endoscopic stenting, or photodynamic therapy. Only rarely clean margins and local lymph node involvement are encountered, and 5-year recurrence-free survival rate is 65%. In patients with gallbladder polyps over 8 mm, cholecystectomy should be performed. Recent tissue markers such as KRAS or p53 in bile are under investigation for screening and diagnosis of cholangiocarcinoma [5]. Gene alterations might affect pathways such as P13K/AKT/mTOR, RAS/RAF/MEK/ERK (KRAS and BRAF), and tyrosine kinase receptor signaling (ERBB2 and FGFR2). Thus, molecular testing is gaining importance due to the potential benefit or target therapies [2,11].

Colonic dysplasia or carcinoma (right-sided colon neoplasia is more frequent) are possible complications of PSC and IBD patients and are diagnosed by colonoscopy with biopsies, preferably with dye-based chromoendoscopy to guide the biopsies [1,12]. Therefore, surveillance in PSC and IBD is annual colonoscopy with biopsy, especially after liver transplantation. Otherwise, a colonoscopy with four-quadrant biopsies from all colonic segments and the ileum should be performed at the moment of diagnosis in patients with PSC, and then, the exam should be repeated every 3–5 years if there is no evidence of colitis. Any dysplastic lesion should be removed endoscopically. However, in case of incomplete or impossible reception, dysplasia in the surrounding mucosa (examined with chromoendoscopy-guided biopsies), invisible high-grade dysplasia, or adenocarcinoma, then proctocolectomy is mandatory [13]. 

Complications associated with cirrhosis and portal hypertension can be encountered (ascites, encephalopathy, varices (encountered in 7–36% of cases with PSC), variceal bleeding, secondary bacterial peritonitis, and hepatocellular carcinoma), and peristomal varices in patients who have undergone proctocolectomy and ileostomy for IBD are associated with the disease [14].

## 8. Management of PSC

Since there is no curative treatment for PSC except for liver transplantation, one should focus on treating and preventing the complications of the disease. More studies are needed to ascertain the role of farnesoid X receptor agonists, norUDCA, bile salt transporter protein inhibitor, antibiotics, and monoclonal antibody blockade of receptors in PSC [7,11].

### 8.1. Medical Treatment of Underlying Disease

#### 8.1.1. Ursodeoxycholic Acid and nonUDCA

Studies have shown that Ursodeoxycholic acid (UDCA) is no longer indicated for routine use in newly diagnosed PSC [5]. Ursodeoxycholic acid (UDCA), a hydrophilic acid, remains the most studied drug in PSC, with an effect on the cholestatic symptoms due to the protective effect against the cytotoxic bile acids, bile acid-induced apoptosis, and its antioxidant effect, stimulating hepatobiliary secretion. Higher mortality, worse clinical outcomes, and need for liver transplant demonstrate that doses over 28 mg/kg/day should not be used in treating PSC. More studies are needed on treatment in early-stage since most of the data from the literature have been developed on patients with fibrosis or cirrhosis. UDCA does not have a benefit in preventing colorectal cancer or cholangiocarcinoma, though studies are still underpowered in this field [5,15]. Low doses of UDCA have a significant effect on biochemistry panel, though there are no significant differences in the risk of cholangiocarcinoma, liver transplant, or death. One indication for UDCA that remains valid is in patients that are already established on treatment, showing that, when stopped, there is worsening of liver biochemistry and pruritus [5]. The optimal dosage that shows moderate clinical, biochemical, and even histological improvement is with intermediate levels of UDCA of 17–23 mg/kg/day (most physicians use 20 mg/kg/day) [4,16]. One way to verify the response is to reevaluate its response at 6 months: if the phosphatase normalizes or is decreased by a minimum of 40% or if the clinical symptoms regress, then UDCA should not be stopped [3,17,18].

#### 8.1.2. Immunosuppressive Drugs

Even though the immunological aspect of PSC is somewhat understood, glucocorticoids orally or by nasobiliary lavage (including budesonide) were not demonstrated to have a benefit in PSC, taking into consideration their severe adverse effects. Tested treatments in PSC that also failed to show any effect include azathioprine, 6-mercaptopurine, docosahexaenoic acid, cyclosporine and tacrolimus, and methotrexate [19]. Penicillamine was first used in PSC due to the increased serum, urinary, and tissular copper concentration. However, studies show that these increased copper stores are due to secondary cholestasis and that penicillamine is not of use. Anti-TNF agents, more precisely Etanercept and Infliximab, failed to show any benefit. Combination of different immunomodulators and UDCA presented no significant histological and cholangiographic improvement in patients with PSC [4,20].

#### 8.1.3. Antibiotics and Probiotics

Hoping to explain the inflammatory factor in the pathogenesis of PSC, physicians have tried to use different combinations of medication to treat the disease. Antibiotics and probiotics aiming to balance the microbiota and to decrease bacterial toll on the liver started to gain importance. Even though vancomycin alone or in combination with metronidazole or minocycline showed little benefit in a certain meta-analysis, further studies are needed to establish the antibiotherapy role in PSC [5,18,21].

### 8.2. Endoscopic Management

ERCP is the go-to strategy in different situations such as cholangitis, contraindication for MRCP, early PSC, AMA-negative PBC, equivocal biopsy, and dominant strictures. Due to the expansion in the therapeutic opportunities in ERCP, a large number of investigations were possible such as endoscopic sphincterotomy (in difficult cannulation, cholelithiasis), hydrostatic balloon dilatation, nasobiliary tube drainage and saline irrigation (used to prevent recurrent biliary obstruction), biliary stent placement, and ductal sampling (brush cytology and endobiliary biopsies with spyglass) [9]. A dominant stricture was described in 36–50% of cases with PSC [22]. Biliary dilatation is preferred over placing plastic stents when facing dominant stricture after malignancy was excluded [5]. To increase the rate of success, balloon dilatations should be repeated at intervals ranging from 1 to 4 weeks for 2–3 consecutive procedures. Studies have shown that, in treating dominant stricture, plastic stents from 7 to 10 Fr in diameter have shown the best outcome. However, in multiple bilateral dominant strictures or hilar structure that expands in the right or left hepatic duct, one can choose2 stents of 7Fr diameter. Short-term stenting with a mean duration of 11 days is more efficient in the long run, with a lower rate of reintervention than in long-term stenting periods (3 months) [13]. Fully covered self-expandable metallic stents are now used in dominant strictures below the liver hilum in PSC patients [5]. 

The complication rate of diagnostic ERCP in PSC is 2%. Most frequent post-therapeutic ERCP complications are pancreatitis (1–7%), cholangitis, bile duct perforation, and worsening cholestasis. ERCP is of no use in patients with intrahepatic biliary strictures and no dominant stenosis, being at a higher risk for post-ERCP cholangitis. Guidelines show that administrating 100 mg of diclofenac or indomethacin prior or immediately after the procedure and prophylactic pancreatic stenting with a 5Fr plastic stent decreases the overall risk of post-ERCP pancreatitis (PEP). There are known factors that increase the risk of PEP such as precut biliary sphincterotomy, pancreatic guidewire-assisted biliary cannulation, endoscopic balloon sphincteroplasty, pancreatic sphincterotomy, and more than 3 of the following: female sex, cannulation time over 10 minutes, more than 1 pancreatic guidewire passage, non-dilated extrahepatic bile ducts, no chronic pancreatitis, normal serum bilirubin, pancreatic injection, and failure to clear bile duct calculi [1,13]. Antibiotic prophylaxis is essential due to the increased risk of cholangitis, bacteremia, and sepsis after bile duct manipulation when bile drainage was incomplete or obtained with difficulty. In these cases, bile fluid sampling and cultures are essential [4].

### 8.3. Percutaneous Treatment

Percutaneous treatment should be used in PSC patients with dominant strictures and altered anatomy that does not allow ERCP procedures (Roux-en-Y choledocojejunostomy or gastric bypass) or intrahepatic strictures or as a rescue therapy after failed endoscopic access. Taking into consideration the impact on patient’s QoL and the higher risk of complications such as hepatic artery injury, hemobilia, and cholangitis, it is usually considered second-line treatment after ERCP [4].

### 8.4. Surgical Therapy in PSC

Once used by many physicians, biliary reconstruction has lost its importance due to the higher mortality and morbidity in time, mainly in patients with associated cirrhosis. Only in isolated focal extrahepatic strictures and early histologic disease, biliary surgery is the first-line treatment. Otherwise, liver transplant is the only curative treatment in PSC, with this disease remaining the fourth cause of liver transplant in the world. End-stage liver disease (Model for End-stage Liver Disease (MELD) ≥ 15 points or The United Kingdom Model for End-Stage Liver Disease (UKELD) over 49), fatigue, pruritus, and painful fractures that influence the QoL are the most common indications for orthotopic liver transplantation. However, there are special circumstances when patients with PSC are referred to liver transplantation despite low MELD scores, also known as MELD exception points such as recurrent or refractory cholangitis, intractable pruritus, and hilar or peripheral cholangiocarcinoma under 3 cm without evidence of metastasis. Outcomes after liver transplant are favorable with a five-year survival rate as high as 85% due to the young age at diagnosis [3,13]. Immunosuppressive treatment after transplant consists of long-term steroids, a calcineurin inhibitor, and a third agent (azathioprineor mycophenolate) [2]. Complications after liver transplant include recurrent PSC found in 9–20% of patients at 5 years, graft rejection (incidence was lowered once immunosuppressant therapy was developed), hepatic artery thrombosis, bile leak, anastomotic stricture, ABO blood group stricture, biliary tract infection, cholelithiasis, and flares of IBD (due to the immunosuppressant therapy post-transplant) [14,23]. The recurrent disease requires retransplantation in about 50% of cases. In overlap syndrome AIH-PSC, a higher frequency of acute and chronic graft rejection was described (39–71%). Patients with PSC and IBD should have stopped smoking and should be in remission at the moment of surgery to improve outcomes after transplant [5]. Recent studies state that biliary dysplasia diagnosed by brush cytology can now represent an indication for liver transplantation [7].

### 8.5. Novel Therapies

Obethicolic acid, an acid-derived farnesoid X receptor (FXR) agonist, is still in clinical studies to observe its efficacy in PSC. Phase 2a studies show great promise in decreasing alkaline phosphatase values, though pruritus is a major side effect [9,24]. Non-bile acid-derived FXR agonists and FGF19 analogues are also in clinical studies, showing not only the FXR agonistic effect but also lower levels of drug toxicity and adverse reactions (pruritus) [9].

Even though the BEZURSO trial shows the efficacy of bezafibrate UDCA (pan peroxisome proliferator-activated receptor (PPAR) agonist) associated with as second-line treatment in PSC, if UDCA fails, there are no randomized controlled trials to ensure these results. Fenofibrate (PPAR-α agonist), seladelpar (orally PPAR-δ agonist), and elabibranor (dual PPAR-α and PAPR-γ agonist) are currently tested in PSC [9,25].

TGR5 agonists were considered as a potential treatment for PSC and primary biliary cholangitis. However, due to the increased risk of cholangiocarcinoma under TGR5 agonists (stimulating the same pathway), all clinical trials were abandoned [26].

Norursodeoxycholic acid (norUDCA) presents a great safety profile, and a phase 3 trial is in progress to establish its efficacy, since norUDCA presents not only anti-cholestatic effects but also anti-inflammatory and anti-fibrotic properties [9].

Studies on fecal microbiota transplant are scarce, though the impact on microbiota might be beneficial in PSC [9].

Recent studies proposed a hypothesis that vedolizumab (anti-integrin antibody) might be used in patients with PSC and IBD by reducing lymphocyte infiltration, thus reducing hepatocyte and biliary inflammation [27]. Vedolizumab and anti-LOXL2 antibodies clinical showed a modest inhomogenous impact on liver biochemistry; thus, further studies are needed, especially randomized clinical trials. However, Timolumab (anti-VAP1 antibody) is currently in phase 2 clinical trial (BUTEO trial) to ascertain its choleretic and anti-fibrotic effects [9].

## 9. PSC follow-Up 

Follow-up of PSC consists of clinical assessment and monitoring of liver biochemistries every 3–4 months. This way, signs of strictures, tumors, or overlap syndrome will be caught early and appropriately managed. Moreover, variations of MELD scores might appear, and the indication for liver transplant might be reevaluated, especially in suspected cholangiocarcinoma. Screening for cholangiocarcinoma consists of cross-sectional imaging (ultrasound or MRI) and CA 19–9 every 6–12 months. In patients with co-existent IBD, annual colonoscopy with chromoendoscopy-guided biopsies is the standard of care [28]. Patients with PSC should be evaluated for osteoporosis, and if discovered, then they should be treated according to national guidelines. Preconception counseling is mandatory in cirrhotic patients due to the maternal and fetal complications that might occur. If there is evidence of cirrhosis and/or portal hypertension, then screening and, if needed, treatment of varices should be considered. Medical centers with a particular interest in PSC should offer patients the possibility to enter clinical trials [5].

## 10. Special Situations

### 10.1. PSC-AIH Overlap Syndrome

PSC-AIH overlap syndrome is defined by the co-existence of the 2 diseases confirmed histologically, encountered in ~10% of patients with PSC with a response to corticotherapy (less pronounced than in AIH without PSC). Often, these patients are younger than 25 years old with the typical biochemical panel: hepatocytolisis with levels of aminotransferases over 5 times the upper limit of normal and autoantibodies (mentioned before). Similarly, it is important to perform MRCP in younger patients with AIH and elevated alkaline phosphatase 2 times the upper limit of normal (ULN) [4].

### 10.2. PSC in Children

PSC in children is less common than in adults and more frequently associated with high levels of aminotransferases and AIH. Cholangiocarcinoma is rarely encountered as a complication of PSC in children; therefore, there is no need for surveillance [4]. Children with PSC often require liver transplant at an early age with a high disease recurrence in the graft. Moreover, they respond poorly to treatment and have a more aggressive evolution of the disease [5].

### 10.3. Immunoglobulin G4-Related Sclerosing Cholangitis

Immunoglobulin G4-related disease combines cholangitis (IgG4-related sclerosing cholangitis) and pancreatitis (type 1 autoimmune pancreatitis/IgG4-related pancreatitis) and describes strictures not only in the biliary ducts but also in the pancreatic duct. Slight elevation of IgG4 levels (up to 5 mg/L or 4×ULN) can be present in patients with PSC that do not meet criteria for IAC [2]. Levels of IgG4 > 140 mg/dL can suggest IAC. The diagnosis using HISORt criteria (≥ 2 manifestations: elevated serum IgG4 (50–80% of patients), suggestive pancreatic imaging, other organ involvement, and bile duct or ampullary biopsy with over 10 IgG4 positive cells/HPF) is extremely important due to the treatment implications it carries since IAC responds to corticotherapy and immunosuppressive agents [29]. When IgG4-SC is suspected, histological diagnosis confirmation is recommended, showing IgG4-positive lymphoplasmacytic infiltrate. Clinical trials show the beneficial effect of anti-CD20 antibody rituximab, though studies are still limited [4,30].

### 10.4. Small Duct PSC

Small duct PSC, also referred to as “pericholangitis”, presents typical clinical and laboratory tests for the disease but with normal cholangiography and accounts for 5 to 20% of patients with PSC. These patients present better prognosis than classical PSC, even if 12–17% of these patients progress to classical PSC [1]. Percutaneous liver biopsy is mandatory in suspected small duct PSC if high-quality MRC is within normal range and in overlap syndrome with autoimmune hepatitis (AIH). Antibiotherapy is essential before the procedure due to the risk of cholangitis [3].

## 11. Conclusions

Even though the pathogenesis of PSC is still a grey area, multiple studies have begun to show the impact of environmental, autoimmune, and ischemic factors and the importance of targeting specific pathways in treating PSC. Game-changing discoveries will provide more facile ways to diagnose the disease with better prognostic models. Studies on gut microbiota and its impact on PSC are evolving, even though at the moment there is no curative treatment for PSC. UDCA is no longer indicated for treating newly diagnosed PSC. When referring to overlap syndrome AIH-PSC or IgG4-SC, corticotherapy remains an effective treatment option. Screening for cholangiocarcinoma must be emphasized especially in light of new imaging modalities and cytological analysis. Searching for early detection biomarkers in bile and serum will play a great role in the future in improving the outcome for patients with cholangiocarcinoma on PSC. New treatment options are needed, especially focused on molecular-targeted therapy.

## Figures and Tables

**Table 1:** Natural history and clinical features in PSC.

Asymptomatic phase	no clinical symptoms cholangiographic abnormalities normal biochemistry
Biochemical phase	asymptomatic biochemical abnormalities
Symptomatic phase	fatigue pruritus and excoriations jaundice fever, chills, night sweats, and right upper quadrant pain * diarrhea and bloody stools **
Decompensated cirrhosis	jaundice ascites muscle atrophy spider telangiectasias peripheral edema hepatosplenomegaly xanthelasma hepatic encephalopathy variceal bleeding

* These symptoms appear in episodic bacterial cholangitis due to biliary obstruction [2]. ** Even if these symptoms are frequent in PSC associated with IBD, bleeding from portal hypertension should be investigated [4].
